# Long Non-coding RNAs in Myeloid Malignancies

**DOI:** 10.3389/fonc.2019.01048

**Published:** 2019-10-18

**Authors:** Alina-Andreea Zimta, Ciprian Tomuleasa, Iman Sahnoune, George A. Calin, Ioana Berindan-Neagoe

**Affiliations:** ^1^MedFuture - Research Center for Advanced Medicine, Iuliu Hatieganu University of Medicine and Pharmacy, Cluj-Napoca, Romania; ^2^Department of Hematology, Research Center for Functional Genomics and Translational Medicine, Iuliu Hatieganu University of Medicine and Pharmacy, Cluj-Napoca, Romania; ^3^Department of Hematology, Ion Chiricuta Clinical Cancer Center, Cluj-Napoca, Romania; ^4^Department of Experimental Therapeutics, The University of Texas MD Anderson Cancer Center, Houston, TX, United States; ^5^Center for RNA Interference and Non-Coding RNAs, The University of Texas MD Anderson Cancer Center, Houston, TX, United States; ^6^MedFuture - Research Center for Advanced Medicine, Research Center for Functional Genomics and Translational Medicine, Iuliu Hatieganu University of Medicine and Pharmacy, Cluj-Napoca, Romania; ^7^Department of Functional Genomics and Experimental Pathology, Ion Chiricuta Clinical Cancer Center, Cluj-Napoca, Romania

**Keywords:** myeloid malignancies, non-coding RNAs, diagnostic tool, prognostic tools, clinical impact

## Abstract

Acute myeloid leukemia (AML) represents 80% of adult leukemias and 15–20% of childhood leukemias. AML are characterized by the presence of 20% blasts or more in the bone marrow, or defining cytogenetic abnormalities. Laboratory diagnoses of myelodysplastic syndromes (MDS) depend on morphological changes based on dysplasia in peripheral blood and bone marrow, including peripheral blood smears, bone marrow aspirate smears, and bone marrow biopsies. As leukemic cells are not functional, the patient develops anemia, neutropenia, and thrombocytopenia, leading to fatigue, recurrent infections, and hemorrhage. The genetic background and associated mutations in AML blasts determine the clinical course of the disease. Over the last decade, non-coding RNAs transcripts that do not codify for proteins but play a role in regulation of functions have been shown to have multiple applications in the diagnosis, prognosis and therapeutic approach of various types of cancers, including myeloid malignancies. After a comprehensive review of current literature, we found reports of multiple long non-coding RNAs (lncRNAs) that can differentiate between AML types and how their exogenous modulation can dramatically change the behavior of AML cells. These lncRNAs include: H19, LINC00877, RP11-84C10, CRINDE, RP11848P1.3, ZNF667-AS1, AC111000.4-202, SFMBT2, LINC02082-201, MEG3, AC009495.2, PVT1, HOTTIP, SNHG5, and CCAT1. In addition, by performing an analysis on available AML data in The Cancer Genome Atlas (TCGA), we found 10 lncRNAs with significantly differential expression between patients in favorable, intermediate/normal, or poor cytogenetic risk categories. These are: DANCR, PRDM16-DT, SNHG6, OIP5-AS1, SNHG16, JPX, FTX, KCNQ1OT1, TP73-AS1, and GAS5. The identification of a molecular signature based on lncRNAs has the potential for have deep clinical significance, as it could potentially help better define the evolution from low-grade MDS to high-grade MDS to AML, changing the course of therapy. This would allow clinicians to provide a more personalized, patient-tailored therapeutic approach, moving from transfusion-based therapy, as is the case for low-grade MDS, to the introduction of azacytidine-based chemotherapy or allogeneic stem cell transplantation, which is the current treatment for high-grade MDS.

## Background on Myeloid Malignancies

Hematopoiesis is the complex process of unidirectional and continuous formation and release of blood cells into circulation. Normal hematopoiesis is polyclonal and takes place in the hematogenous bone marrow (BM) (1–3). Pluripotent stem cells have the capacity to differentiate into multiple cell lineages (4–7), and this differentiation is irreversible during normal human physiology. The production of hematogenous BM is impressive, forming about 10^10^ erythrocytes and 10^8^-10^9^ leukocytes every hour, regulated by numerous cytokines for the maintenance of lineages under normal parameters (8–10). When in need, production can increase up to seven times normal levels. Pluripotent stem cells differentiate into myeloid and lymphoid multipotent stem cells that further differentiate into cells oriented on a lineage, called colony forming units (CFU). This name depicts their capacity to produce colonies *in vitro* in the presence of growth factors, also called progenitors (11, 12).

**Figure 1:**
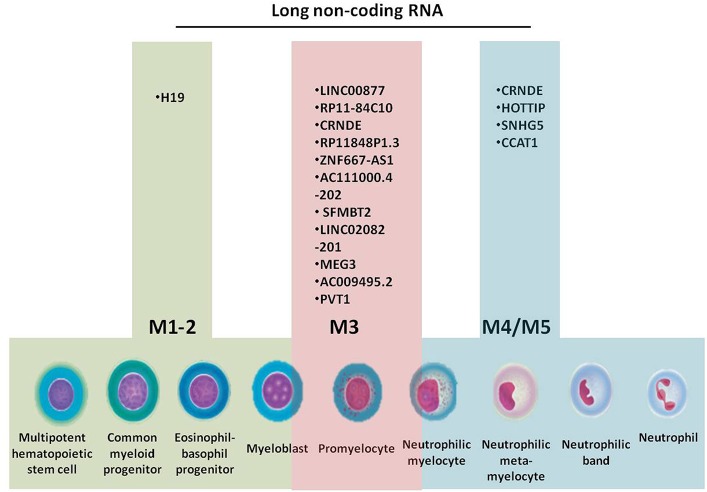
Granulopoiesis, the generation of myeloid cells, French-American-British (FAB) classification of AML and the specific long non-coding RNAs for each AML subtype.

All cells of the BM, including progenitors, precursors, mature cells and stromal cells, interact in a complex manner through cytokines, messenger RNAs (mRNAs), microRNAs, and other non-coding RNAs (ncRNAs) (13–16), by targeting genes responsible for cell proliferation, differentiation, methylation, and acquisition of resistance to therapy. Granulopoiesis is the process that gives rise to elements of the granulocyte series: segmented neutrophils, eosinophils, and basophils. Acute myeloid leukemia (AML) develops in different stages of granulopoiesis, each with a specific set of long non-coding RNAs (lncRNAs) (Figure 1). The progenitors of this series are the myeloid stem cell and the granulo-monocytic colony forming unit, which are not morphologically identifiable (17, 18).

Non-coding RNAs are small RNA molecules that are not translated into a protein (19), and have the potential of bringing new insights in the clinic for diagnosis, prognosis and therapy. DNA is a molecule composed of around 3 billion base pairs, out of which only 5–10% are transcribed, with protein-coding genes accounting for <2% of the human genome (20–22). Between 60% and 90% of the human genome is transcribed in non-coding transcripts, these transcripts being further divided into two categories depending on their primary role: either housekeeping or regulatory genes (23). Regulatory genes include microRNAs (miRNAs), circular RNAs and lncRNAs (24, 25) involved in regulation of gene transcription and translation, along with key cellular mechanisms such as differentiation, proliferation, or inflammation. In this manuscript, we aim to provide novel comprehensive insights on the role of lncRNAs in the pathogenesis of myeloid malignancies, illustrating their diagnostic and prognostic potential.

Myeloid precursors are formed by at least four generations of cells between granulo-monocytic CFUs and the mature elements of the series. Young precursors have azurophil granulations, also called primary granulations. Lineage-specific granulations (neutrophil, eosinophil, basophile) appear at the myelocyte stage. All lineages have the same maturation steps, with the only difference occurring in lineage specific granulations (26–29).

**Figure 2:**
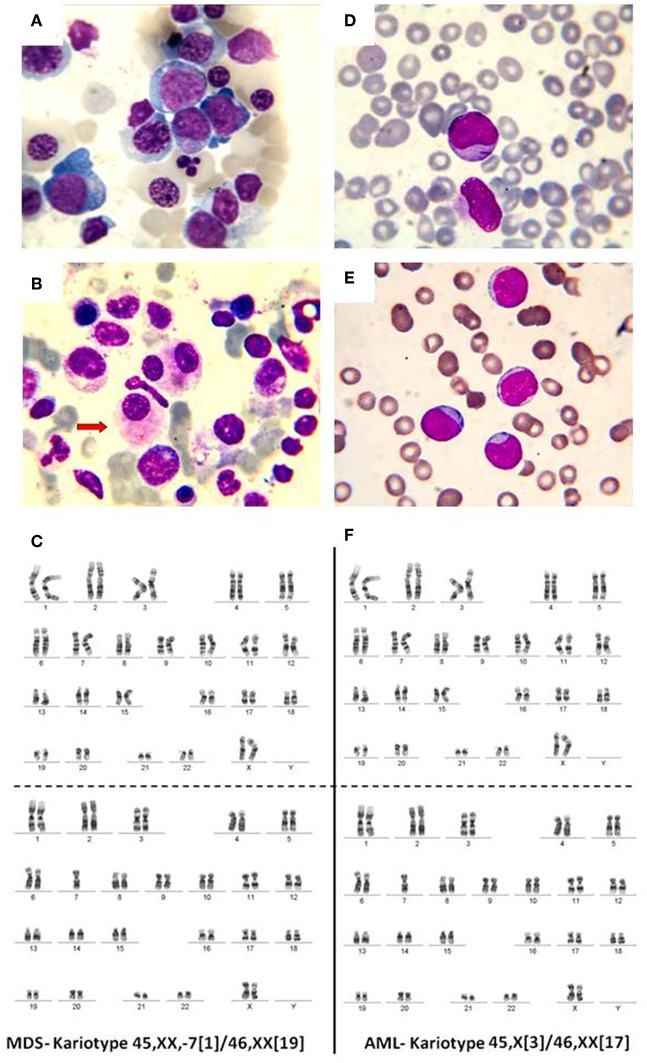
**(A)** Bone marrow, May-Grunwald-Giemsa stain, 1,000x. Myelodysplastic syndrome. Hyperplasic erythroid lineage, megaloblasts, oxyphilic megaloblast with sprouted nuclei, **(B)** myelodysplastic syndrome, dysmegakaryocytopoiesis. **(C)** MDS karyotype classified as intermediate risk. **(D)** Myeloblasts. Acute myeloid leukemia, **(E)** weakly differentiated myeloblasts. Acute myeloid leukemia, **(F)** AML karyotype classified as intermediate risk.

In normal conditions, the BM contains <5% blasts. When a mutation appears in a myeloid progenitor of the BM, the result is the development of a myelodysplastic syndrome (<20% blasts) or an acute leukemia (more than 20% BM blasts). Myelodysplastic syndromes (MDS) are clonal disorders of hematopoietic stem cells, characterized by cytopenia in the context of hyper or normocellular bone marrow, multilineage dysplasia, and frequent chromosomal abnormalities (30–33). The World Health Organization (WHO) classification based on morphology, cytochemistry, immunophenotype, cytogenetics, and clinical examination is described in Table 1 and Figure 2 (34–36).

**Table 1:** Classification of myelodysplastic syndromes.

**Disease**	**Peripheral blood**	**Bone marrow**
Refractory cytopenia with unilineage dysplasia (refractory anemia, refractory neutropenia, refractory thrombocytopenia)	Isolated cytopenia or bicytopenia <1% blasts	Unilineage dysplasia: ≥10% of the cells of one myeloid lineage <5% blasts <15% ring sideroblasts
Refractory anemia with ring sideroblasts	Anemia 1% blasts	Dysplastic erythroid series <5% blasts ≥15% ring sideroblasts
Refractory cytopenia with multilineage dysplasia	Cytopenia <1 % blasts <1 × 10^3^/μl monocytes without Auer bodies	Dysplasia ≥10% cells in ≥2 myeloid lineages <5% blasts Without Auer bodies <15% ring sideroblasts
Refractory cytopenia with multilineage dysplasia and ring sideroblasts	Cytopenia <1% Blasts <1 × 10^3^/μl monocytes without Auer bodies	Dysplasia ≥10% cells in ≥2 myeloid lineages <5% blasts Without Auer bodies >15% ring sideroblasts
Refractory anemia with excess blasts 1	Cytopenia <5% blasts Without Auer bodies ±1 × 10^3^/μl monocytes	Uni or multilineage dysplasia 5–9% blasts Without Auer bodies
Refractory anemia with excess blasts 2	Cytopenia 5–19% blasts ±Auer bodies ±1 × 10^3^/μl monocytes	Uni or multilineage dysplasia 10–19% blasts ±Auer bodies
Unclassified myelodysplastic syndrome	Cytopenia <1% blasts	Dysplasia in <10% of the cells of one or more myeloid lineages associated with a cytogenetic abnormality <5% blasts
Myelodysplastic syndrome associated with isolated 5q-	Anemia Usually: normal or elevated thrombocytes <1% blasts	Elevated megakaryocytes with hypolobulated nuclei <5% blasts Unique cytogenetic abnormality: 5q- Without Auer bodies

## Cytogenetics in the Clinic for Myeloid Malignancies

Recurrent cytogenetic abnormalities are considered diagnostic for myelodysplastic syndrome, in the presence of cytopenia of unknown etiology and in the absence of myelodysplastic changes. Morphologic changes that are characteristic for myelodysplastic syndrome are described in Table 2 (37–39).

**Table 2:** Recurrent abnormalities in myelodysplastic syndromes.

**Unbalanced abnormalities**	**Balanced abnormalities**
−7 or del (7q)	*t*(11;16)(q23;p13.3)
−5 or del (5q)	*t*(3;21)(q26.2;q22.1)
I (17q) or t (17p)	*t*(1;3)(p36.3;q21.1)
−13 or del (13 q)	*t*(2;11)(p21;q23)
Del (11q)	inv(3)(q21q26.2)
Del (12p) or t (12p)	*t*(6;9)(p23;q34)
Del (9q)	
(X)(q13)	

Depending on genetic background, MDS can either respond to standard therapies, or can evolve into an acute leukemia (40–43). Acute leukemias are caused by mutations in the unipotent or pluripotent stem cells from the bone marrow that yield an overproduction of immature cells and inhibition of normal hematopoiesis, resulting in anemia, thrombocytopenia, and neutropenia (42, 44–47). The type of malignant cell is defined according to the lineage, whether it is myeloid, lymphoid or mixed. Acute leukemias also occur when a hematopoietic stem cell acquires a sequence of mutations that confer an advantage of clonal proliferation (43, 48–52). The incidence of AML increases with age and represents 80% of adult leukemia (53) and 15–20% of childhood acute leukemiac (54). Congenital leukemia is a rare disease that is acquired in the neonatal period (45). For AML, the symptomatology results from bone marrow infiltration by the malignant cell, which inhibits normal hematopoiesis. Leukemic cells are not functional; thus, the patient develops anemia, neutropenia, and thrombocytopenia, leading to fatigue, recurrent infections, and hemorrhage.

A common characteristic of acute leukemia is the hypercellularity of the bone marrow, with more than 20% blasts at the time of diagnosis. Blast subtype represents the principal diagnostic criteria between acute lymphoblastic leukemia and AML. WHO classification of AML divides them into four principal subtypes: AML with recurrent cytogenetic abnormalities, AML with dysplasia, AML associated with therapy and myelodysplastic syndrome, and the “not otherwise specified” AML (34).

Cytogenetics and molecular biology are of key importance in myeloid malignancies, as previously shown by several groups including ours (55–58). In AML with recurrent cytogenetic abnormalities, these very recurrent cytogenetics represent the most important trait, as their names clearly describes them (55–57). The best prognostics are associated with *t*(8;21) (q22;q22) and inv(16)(p13;q22), where a high percentage of patients achieve complete remission (58, 59). Patients with AML are routinely profiled for the presence of mutations in FLT3, NPM1, CEBPA, and, more recently, TP53 (59–61). According to the German-Austrian Acute Myeloid Leukemia Study Group, coordinated by Dohner et al., the genotype of mutant NPM1 without FLT3-ITD, the mutant CEBPA genotype, and younger age were each significantly associated with complete remission. The benefit of the transplant was limited to the subgroup of patients with the genotype FLT3-ITD or the genotype consisting of wild-type NPM1 and CEBPA without FLT3-ITD.

AML with dysplasia is associated with at least two dysplastic lineages and the leukemic clone develops following a previous MDS clone. Dyserythropoiesis is described as multinucleated erythroblasts, magaloblastosis, cytoplasmic vacuoles or karyorrhexis (62). The leukemic clone develops as an evolutionary hallmark of MDS and patients generally present with a reduction in the number of erythrocytes, neutrophils and thrombocytes (63, 64). Because of frequent pancytopenia, the prognosis is generally bad. Several predictors for transformation have been identified and include mutations of genes in growth signaling pathways (NRAS, KRAS, PTPN11, FLT3), mutations in genes more commonly observed in AML (NPM1, WT1, IDH2), and certain cytogenetic abnormalities (monosomy 7, complex karyotype, loss of 17p). Gene expression profiles that divide MDS into two major categories identify a progenitor gene signature subtype associated with a high risk of AML transformation (65).

Therapy-related MDS (t-MDS) and therapy-related AML (t-AML) are considered one entity in therapy-related myeloid malignancies because of their similar pathogenesis, rapid progression from t-MDS to t-AML, and their equally poor prognosis. A small percentage of patients present with favorable risk fusion genes, whereas 50% have adverse cytogenetics. The most frequent molecular aberration in t-AML and t-MDS affects TP53 (33%). The selection of pre-existing treatment-resistant hematopoietic stem cell clones with the TP53 mutation has been shown as an important mechanism in the development of t-MNs and explains the high frequency of TP53 mutations in these patients (66, 67).

## Current Research on Long Non-Coding RNAs in Myeloid Malignancies

As previously mentioned, ncRNAs are transcripts that do not possess protein-coding activity, but regulate cell behavior through interactions established at the DNA, RNA, or protein levels. Due to their versatility, the ncRNAs are often proposed as diagnostic and prognostic tools (68, 69) or as part of combined therapy in cancer (70–72). The role of ncRNAs has been extensively analyzed during the past decade, with the focus being on miRNAs (73–77). LncRNAs are a class of transcripts with sequence lengths of more than 200 nucleotides (78, 79). They are involved in various molecular processes, such as chromatin interaction (80), modulating gene transcription through binding the promoter region (81), acting as a competing endogenous RNAs (ceRNAs) for microRNAs (82), interacting with the ribosome and thus interfering in translation as well as interacting with various proteins (83, 84) and determining cellular localization (84). Non-coding transcription across antisense strands of genes is a universal mechanism for both yeast and vertebrates. The antisense transcription stimulates nucleosome occupancy and acetylation of histone proteins, but does not always alter the transcription of protein-coding genes. Antisense transcription has a high level of histone turnover and makes genes more susceptible to changing signals due to a wide range of chromatic configuration. This is specific to eukaryotic cells and allows these cells to adapt to external environmental stress (85–87). Due to this supportive evidence, the role of lncRNAs in hematological malignancies is being investigated and numerous studies offer compelling evidence for the correlation between altered lncRNA expression and various clinico-pathological characteristics of leukemia patients.

SBF2-AS1, DANCR, LINC00239, LINC00319, LINC00265, LEF1-AS1, and ZFAS1 are lncRNAs found to be overexpressed in AML (88–94). *In vitro*, the inhibition of SBF2-AS1 lncRNA causes leukemia cells to undergo apoptosis and cell cycle arrest. This lncRNA sponges the tumor suppressor miR-188-5p. The lncRNA DANCR is downregulated in undifferentiated hematopoietic progenitors, both malignant and BM-derived normal stem cells (89, 95, 96), the CD34-positive leukemic stem cells, as well as in normal BM-derived. However, for normal stem cells, siRNA-mediated knock out of DANCR does not affect normal hematopoiesis. In AML cells, DANCR silencing leads to impaired AML progression through inhibited self-renewal capacity and dormancy of leukemia cells. *In vivo* experiments also showed that the siRNA treatment leads to increased overall survival. This lncRNA acts by activating the c-Myc transcription factor through the WNT signaling pathway (89).

Linc00239 regulates chemoresistance to doxorubicin and exerts a protective effect against apoptotic cell death, promotes cell viability, cell cycle distribution, colony formation and migration. This lncRNA leads to mTOR/AKT activation (90). The overactivation of SOX61 in AML leads to increased cell proliferation and inhibited cell apoptosis in KG-1 cells and THP-1 cell (97). LINC00265 causes G0/G1 cell cycle arrest, decreased proliferative rates, apoptosis, and reduction of migratory capabilities and is involved in the phosphorylation of PI3K/AKT (92).

Some lncRNAs act as tumor suppressors in AML. H22954 is a novel lncRNA, downregulated in the bone marrow of AML patients. Its decreased expression is linked to a higher risk of relapse. H22954 expression inhibits AML cell proliferation *in vitro* and its overexpression leads to cell apoptosis. *In vivo*, it inhibits tumor growth in a mouse xenograft model as it interacts with the 3′UTR BCL2, a well-known antiapoptotic oncoprotein (98).

In AML with a monocytic/monoblastic phenotype, HOTTIP and LINC00152 are overexpressed specifically in the bone marrow microenvironment of patients with AML FAB classification M5. *In vitro* experiments have proven that the inhibition of LINC00152 leads to impaired proliferation of leukemia cells and cell cycle arrest (99, 100). LINC00152 decreases the self-renewal capacity of leukemia cells (99), whereas HOTTIP acts by sponging the tumor suppressor miR-608 and the consequent overstimulation of the oncogene DDA1 (DET1- and DDB1-Associated Protein 1) (100). LINC00152 has a similar molecular activity, by sponging miR-193a and indirectly increasing the expression of Cyclin Dependent Kinase 9 (CDK9). A subtype of AML is acute promyelocytic leukemia (APL), the former FAB M3 AML, with a particular genetic background and therapy protocol, based on the use of retinoic acid (101–103). In APL, the overexpression of ENST00000484765, ENST00000509010, and ENST00000416842 translates into a negative overall survival with a higher percentage of cancer stem cells present in the bone marrow, whereas the overexpression of ENST00000505646, lnc-SFMBT2-4 is translated into a better survival rate for the patients (104). Therefore, the expression analysis of these lncRNAs could be incorporated into current clinical practice (104).

TUG1 (105, 106), SNHG5 (107), LINC00926, LRRC75A-AS1, FAM30A (108), IRAIN (109), ENSG00000260257, and ENSG00000236537 (110) can stratify AML patients into those with higher survival rates or lower survival rates, according to their expression level.

UCA1 is upregulated in the AML cell line HL-60, and its inhibition decreases cell viability, migration and invasion capacity (111), while surpassing chemoresistance (112). This lncRNA targets two tumor suppressor microRNAs, namely miR-126 in AML (111) and miR-125A in *de novo* AML (112).

H19 is a potential biomarker of AML response to therapy, being downregulated in CR. It has the ID2 gene as a potential downstream target. This lncRNA impairs apoptosis and promotes cell proliferation in AML (113). MALAT1 is a well-known lncRNA with oncogenic potential in solid cancers. In AML, a number of studies have reaffirmed its oncogenic role. MALAT1 correlated with the expression of NTRK3 gene can differentiate between patients carrying various mutations. For instance, patients with *t*(15;17) and the PML-RARA fusion gene have increased co-expression of MALAT1 and NTRK3; patients with *t*(8;21) exhibit an overexpression of MALAT1 and underexpression of NTRK3. As follows, in the clinical setting AML patients carrying the ETV6-NTRK3 gene fusion and associated MALAT1 overexpression can exhibit a better therapeutic response to NTRK3 inhibitor, AZ-23 (114).

HOTAIR is another oncogenic lncRNA with well-established roles in various tumors. The expression of HOTAIR is upregulated in AML cell lines (HL-60, K562) (115, 116) and in peripheral or bone marrow mononuclear cells from AML patients. It stimulates AML progression, by promoting a higher number of blast and stimulated cell proliferation (115–117).

ZEAS1 is upregulated in AML cell lines, and its knockdown lowers cell proliferation, induces apoptosis and cell cycle arrest in the G0/S phase (118). CCDC26 is upregulated in AML cell line K562, where it stimulates cell division by impairing the translation of KIT proteins (119). Further details on the role of lncRNAs in leukemia are found in Table 3.

**Table 3:** Long non-coding RNAs in myeloid malignancies.

**Name of lncRNA**	**Expression**	**Targets**	**Type of sample**	**Effect**	**References**
HOTTIP	UP	HOTTIP/microRNA-608/DDA1 axis	AML cell lines, bone marrow	Proliferation and cell cycle progression	(95)
HOTAIR	UP	N/A	Bone marrow and peripheral blood mononuclear cells, AML cell line	Associated with higher white blood cell and BM blast counts, decreased overall survival, increased cell proliferation	(109, 110)
	UP	miR-193a	Bone marrow mononuclear cells, AML cell line	Maintenance of the malignant phenotypes	(111)
	UP	p15	AML cell lines, umbilical cord blood, murine bone marrow progenitor cells	Decreases proliferation and colony of in AML CD34^+^ progenitor cells	(114)
SBF2-AS1	UP	miR-188-5p	AML cell lines	SBF2-AS1 inhibition induced AML cells apoptosis and arrested AML cells in G0/G1 phase	(83)
CCDC26	UP	Translation of KIT protein	AML cell line	Inhibition slows cell proliferation	(113)
	UP	N/A	Bone marrow mononuclear cells	Differentiation between patients with a specific FAB class (M3) or cytogenetic risk	(115)
PVT1	UP				
	UP	N/A	Murine bone marrow samples	Specific for leukemia cells	(116)
	UP	c-Myc	Bone marrow	adverse prognosis and shorter overall survival; higher levels in *t*(8;21) positive—AML by 5.3-folds compared to *t*(8;21) negative group	(117)
CCAT1					
	UP	miR-155	Peripheral blood mononuclear cells, AML cell line	Repressed monocytic differentiation and promoted cell growth in AML M4 and M5	(118)
DANCR	UP	c-MYC (and the whole WNT signaling pathway)	Bone marrow and peripheral blood samples	Impaired AML progression, through inhibited self-renewal capacity and dormancy of leukemia cells	(84)
LINC00239	UP	mTOR, AKT phosphorylation	AML cell lines	Regulates chemoresistance to doxorubicin and exerts a protective effect against apoptotic cell death, promotes cell viability, cell cycle distribution, colony formation and migration, knock out does not have significant therapeutic effect, but induced overexpression was much worse than the control	(85)
CYTOR^a^	Up	miR-193a, CDK9	Bone marrow samples, AML cell lines	Suppresses the proliferation, accelerates the apoptosis, and induces the cycle arrest of, decreased number of colonies	(94)
RP11-395P13.6-001	UP	N/A	Bone marrow samples	Independently predicted poor OS, these are especially found in stem cells, AML-M3, depending on the risk factor, high risk vs. low risk group	(98)
AP001042.1					
LINC02082					
	UP	N/A	AML cell lines, Bone marrow samples	Promoted cell proliferation and inhibited cell apoptosis in KG-1 cells and THP-1 cells, pediatric AML patients	(92)
LINC00319	UP	Stability of SIRT6	AML cell lines	Its silencing represses the growth of AML cells	(86)
LINC00265	UP	Phosphorylation of PI3K and Akt	Bone marrow samples and serum, AML cell lines	G0/G1 cell cycle arrest, decreased proliferative rates, apoptosis, reduction of migratory capabilities	(87)
LEF1-AS1	UP	p21 and p27	Bone marrow samples	Inhibited proliferation, less cell divisions, no difference in apoptosis levels	(88)
PANDAR	UP	N/A	Bone marrow mononuclear cells	Upregulation in non-M3-AML and cytogenetically normal AML	(88)
TUG1	UP	N/A	Bone marrow samples and AML cell lines	Correlated with poor risk stratification, up-regulated especially in M1-AML patients, TUG inhibition decreases cell viability, increased apoptosis	(99)
		AURKA—coexpression	Bone marrow samples and AML cell lines	Decreased survival rate, can differentiate between different risk categories, increased proliferation rate, decreased apoptosis	(100)
CDKN2B-AS1^b^	UP	AdipoR1	Bone marrow samples	Promotes cell senescence and apoptosis	(119)
SNHG5	UP	N/A	Bone marrow samples and plasma	Poorer prognosis in AML (M4–M5)	(101)
LINC00926	UP	322 potential mRNA targets	TCGA data	Favorable survival	(102)
SNHG29^c^					
FAM30A	UP			Poor survival	
UCA1	UP	miR-126	AML cell lines	Knockdown inhibited cell viability, migration, and invasion, while stimulating apoptosis	(105)
	UP	miR-125A	AML cell lines	Increase of chemoresistance of pediatric AML Adriamycin-resistant	(106)
H19	UP	Potential downstream gene ID2	TCGA and GEO, AML cell line, bone marrow samples	Significantly shorter OS, pro-proliferative and anti-apoptotic effects in leukemia cells	(107)
	UP	miR19a/b	Bone marrow samples, AML cell line	Knockdown inhibited AML cell proliferation, colony formation in AML-M2	(120)
LINC00899	UP	N/A	Bone marrow mononuclear cells	Differences between AML and iron deficiency anemia (IDA) sample	(121)
RP11-305O.6					
RP11-222k16.2					
	UP	Eomes	TCGA data	LncRNA-mediated dysregulation of Eomes, blocking of NK cell differentiation	
MALAT1	UP	Co-expression with NTRK3	AML cell line	NTRK3 HIGH/MALAT1 HIGH patients carry *t*(15;17) mutation, and PML-RARA fusion, while MALAT1 HIGH /NTRK3 LOW carry *t*(8;21) mutation	(108)
NEAT1	UP	C/EBPβ bind to the promoter of lncRNA NEAT1	AML cell line	Knockdown of C/EBPβ impairs ATRA-induced upregulation of NEAT1 in AML-M3	(122)
PILNA	UP	N/A		Expressed in hematopoietic progenitors	
LNC_177417	UP	N/A	Murine bone marrow samples	Maintenance of cell stemness	(116)
LNC_104449					
AC009495.2	UP	N/A	TCGA data and bone marrow samples	Expression specific for AML-M3 subtype	(123)
MEG3					
	DOWN	miR-22-5p	AML cell line	Stimulates cell reproductive capacity	(124)
TET2		miR-22-3p			
CASC15	UP	Targets the overexpression of SOX4, through its interaction with YY1	AML cell lines	Overexpression opposes cellular proliferation and promotes myeloid bias *in vivo* in AML with RUX translocation	(125)
XLOC_109948	UP	N/A	Bone marrow samples, AML cell line	Reduces overall survival, apoptosis resistance in NPM1-mutated AML	(126)
AL035071.1	UP	Co-expression with MAPRE	Bone marrow mononuclear cell	Worse prognostic, shorter overall survival	(104)
RP11-732M18.3	UP	Co-expression with TULP4		Worse prognostic, shorter overall survival	
MIR9-3HG	UP	N/A		Overexpressed in BM stem cells vs. differentiated cells	
LINC00467	UP			Overexpressed in AML cells vs. BM stem cells	
ZFAS1	UP	N/A	AML cell lines	Inhibition leads to decreased cell proliferation, apoptosis induction, and cell cycle arrest	(112)
H22954^d^	DOWN	BCL2, miR-5095, and miR-619-5p	AML cell lines, Bone marrow	Increased risk of relapse, H22954 expression inhibits AML cell proliferation *in vitro*, overexpression of this lncRNA leads to apoptosis, H22954 expression inhibits tumor growth in a mouse xenograft model, H22954 interacts with the BCL2 3′UTR	(93)
LINC00504	UP	N/A	TCGA data	Increased peripheral blast	(127)
CRNDE^e^				Bone marrow blasts	
	UP	N/A	Bone marrow samples and AML cell lines	Inhibition lowers proliferation and self-renewal capacity, while increasing apoptosis	(128)
	DOWN	N/A	TCGA data and bone marrow samples	Expression specific for AML-M3 subtype	(123)
LINC00877					
RP11-84C10.2					
RP11-848P1.3					
ZNF667-AS1					
RP11-704M14.1	DOWN		Bone marrow samples	Predicts better overall survival, these are especially found in stem, cells, AML-M3, depending on the risk factor, high risk vs. low risk group	(98)
SFMBT2-4:1					
IRAIN	DOWN	N/A	Bone marrow samples	Poor prognostic factor for non-M3 AML	(103)

*Results from literature review*.

a *Also known as LINC00152*.

b *Also known as ANRIL*.

c *Also known as LRRC75A-AS1*.

d *De novo discovery, Uncharacterized LOC100996457*.

e *CRNDE gene can be transcribed into protein coding or non-coding transcripts, the lncRNA variant is also known under the name: LINC00180*.

The search for lncRNAs biomarkers for AML can yield negative results. For instance, Sayad et al. have tried to analyze the expression level of FAS antisense RNA 1 (FAS-AS1) in the peripheral blood of AML patients vs. healthy controls. The study found no significant change in expression between the two groups, even though FAS has been found to play key roles in cellular apoptosis (120). In a study involving an Iranian population, it was proven that HOTAIR is not differentially expressed in the peripheral blood from healthy vs. AML patients (121). Several lncRNAs were reported to have both oncogenic and tumor suppressor roles in AML. On the one hand, MEG3 is upregulated in APL (122), whereas in AML cell lines, it was found to function as a tumor suppressor (123). On the other hand, TCGA analysis revealed that CRINDE is downregulated in APL (122) and upregulated in bone marrow stem cells (124), bone marrow samples and AML cell lines (125). This could be attributed to the fact that the AML cell lines were harvested from patients with other AML subtypes or due to differences in gene expression analysis.

## Long Non-Coding RNAs in Myeloid Malignancies—Analysis of TCGA Data

In the present manuscript we also present a TCGA data analysis on myeloid malignancies. The TCGA data on the clinical characteristics of the patients and RNASeq counts was downloaded from the GDC TCGA Acute Myeloid Leukemia (LAML) cohort, which included 151 patients (https://xenabrowser.net/datapages/).

From the RNAcentral database (https://rnacentral.org/search?q=RNA), we downloaded the full set of human lncRNAs found in the Ensembl database. A total of 27.969 Ensemble ID codes for lncRNAs were retrieved and using a Venn diagram (http://bioinfogp.cnb.csic.es/tools/venny/), we identified the list of lncRNAs from the TCGA dataset, reaching a total of 275 lncRNAs. We ran a wide-range gene expression analysis with an online gene expression analysis tool based on the Jupyter Notebook in Python (126).

**Figure 3:**
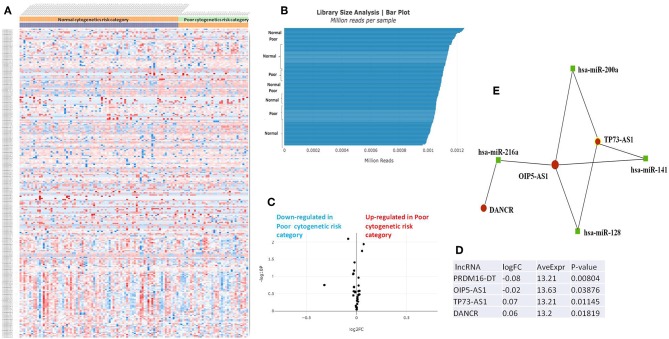
**(A)** Heat map comparing the expression level of lncRNAs in Poor vs. Normal risk category. **(B)** Library Size Analysis between TCGA samples belonging to Poor or Normal risk category. **(C)** Volcano plot analysis of lncRNA expression differentiating Poor vs. Normal risk category. The set FC was at 1 and *p* value at <0.05. **(D)** Table of lncRNAs with significant different expression between the Poor and Normal risk category, their fold change, average expression and *p*-value. **(E)** LncRNA-microRNA interaction network analyzing the common ceRNA activities of the lncRNAs with different expression between Poor or Normal risk category.

**Figure 4:**
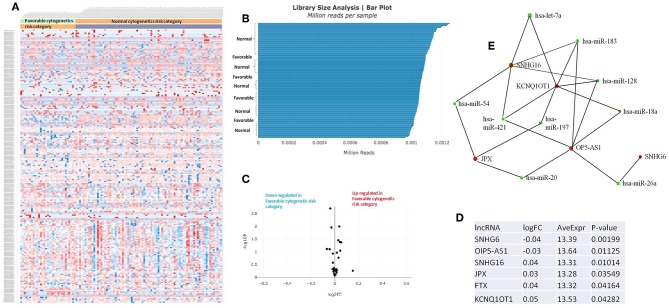
**(A)** Heat map comparing the expression level of lncRNAs in Favorable vs. Normal risk category. **(B)** Library Size Analysis between TCGA samples belonging to Favorable or Normal risk category. **(C)** Volcano plot analysis of lncRNA expression differentiating Favorable vs. Normal risk category. The set FC was at 1 and *p* value at <0.05. **(D)** Table of lncRNAs with significant different expression between the Favorable and Normal risk category, their fold change, average expression, and p-value. **(E)** LncRNA-microRNA interaction network analyzing the common ceRNA activities of the lncRNAs with different expression between Favorable or Normal risk category.

**Figure 5:**
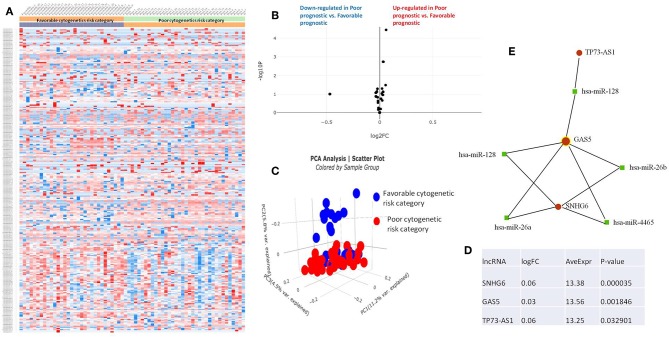
**(A)** Heat map comparing the expression level of lncRNAs in Favorable vs. Poor risk category. **(B)** Volcano plot analysis of lncRNA expression differentiating Poor vs. Normal risk category. The set FC was at 1 and *p* value at <0.05. **(C)** Principal component analysis (PCA) comparing the Favorable risk category vs. Poor risk category. **(D)** Table of lncRNAs with significant different expression between the Poor and Normal risk category, their fold change, average expression and *p*-value. **(E)** LncRNA-microRNA interaction network analyzing the common ceRNA activities of the lncRNAs with different expression between Poor or Normal risk category.

The RNASeq data showed an evenly distributed million reads per sample across the three analyzed cytogenetic risk groups: Figure 3 compares the expression level of lncRNAs in poor vs. normal risk category patients, Figure 4 depicts favorable vs. normal risk category, whereas Figure 5 depicts favorable vs. poor risk patients.

The heatmaps comparing the expression of lncRNAs between the poor vs. normal risk category, favorable vs. normal risk category, poor vs. favorable risk category, showed a heterogeneous distribution of lncRNA expression. By calculating the fold change between the peripheral blood gene expressions between each compared category, we were able to identify a total number of 10 lncRNAs that were significantly differentially expressed between each of the two analyzed groups.

**Figure 6:**
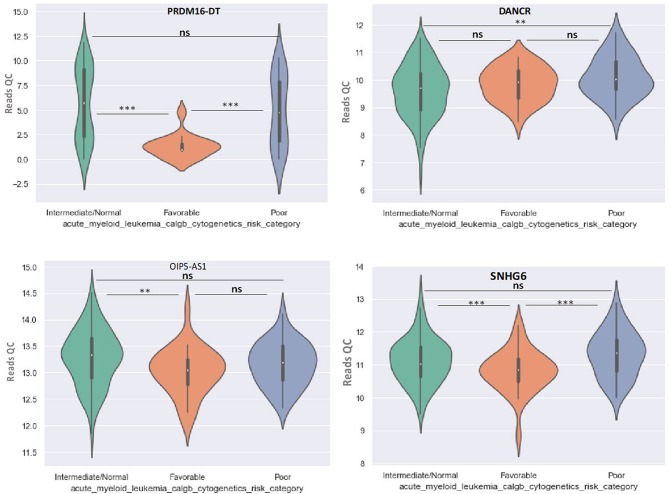
Violin plot of lncRNA with different expression between Normal, Favorable, and Poor risk category for PRDM16-DT, DANCR, OIP5-AS1, SNHG16 lncRNAs. ^**^ represents statistically significant data. ^***^ represents very highly statistically significant data.

Principal Component Analysis (PCA) revealed a significant clustering of lncRNA expression in the case of favorable vs. poor prognostic cytogenetic risk category (Figure 5). With the help of the online tool miRNet (https://www.mirnet.ca/miRNet/faces/home.xhtml), we were able to construct three lncRNA-microRNA interaction networks that have shown common ceRNA activity between the lncRNAs with differential expression between the analyzed groups. To better illustrate the difference in expression between the three groups, we developed violin plots in the Jupyter Notebook. PRDM16-DT has a highly statistically significant (*p* <0.001) downregulated expression in the favorable risk category in comparison with normal or poor cytogenetic risk categories (Figure 6).

DANCR is upregulated in poor prognostic cytogenetic patients in comparison to the intermediate/normal risk category, while the difference between favorable and poor or normal risk is not significant (Figure 6).

**Figure 7:**
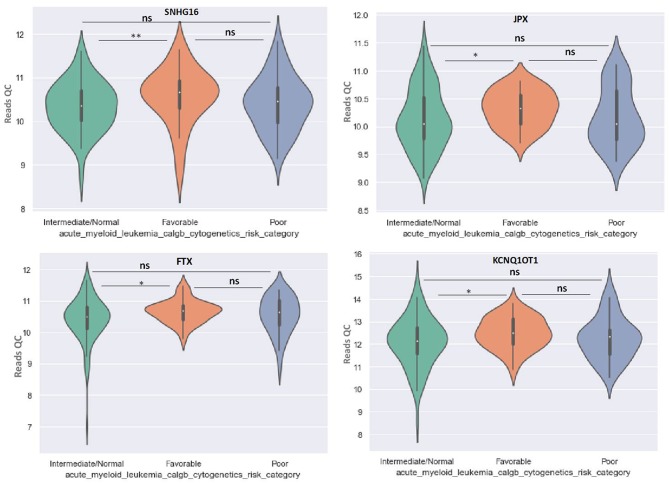
Violin plot of lncRNA with different expression between Normal, Favorable, and Poor risk category for SNHG16, JPX, FTX, and KCNQ1OT1 lncRNAs. ^*^ represents statistically significant data. ^**^ represents very highly statistically significant data.

OIP5-AS1 is down-regulated in favorable risk categories in comparison with normal risk category (*p* < 0.01). The difference in expression between the other two categories is not significant (Figure 6). SNHG16, JPX FTX, and KCNQ1OT1 are significantly upregulated in the cytogenetically favorable risk category. The difference in expression between the other two categories is not significant (Figure 7).

**Figure 8:**
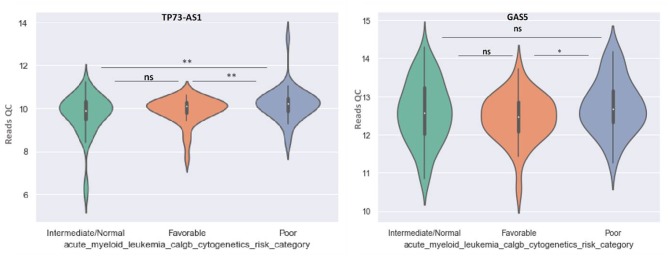
Violin plot of lncRNA with different expression between Normal, Favorable, and Poor risk category for TP73-AS1 and GAS5 lncRNAs.

GAS5 can differentiate between favorable and poor risk categories, being overexpressed in patients with poor cytogenetic prognostic (Figure 8). TP73-AS1 is upregulated in poor risk category in comparison with favorable and normal risk categories (*p* < 0.01) (Figure 8).

There is published data on analysis made in AML cell lines after long-term exposure to chemotherapeutics or bone marrow analysis post-treatment. The lncRNA named urothelial carcinoma-associated 1 (UCA1) is generally overexpressed in AML and it was proven that acquired chemoresistance to adriamycin in HL60 cells is correlated with increased expression of UCA1. In adriamycin–resistant AML cells UCA1 sponges miR-125a thus releasing hexokinase 2 (HK2) and hypoxia-inducible factor 1α (HIF-1α) from its control. The overexpression of UCA1 in post-treatment AML cells was confirmed by clinical analysis done on pediatric patients (112). Linc00239 is another lncRNA whose increased expression is related to a higher resistance to doxorubicin, as shown by the KG-1 and HL-60 AML cell lines. However, it is involved in the intrinsic resistance to chemotherapy and its expression in acquired chemoresistance was not evaluated (90). HOXA-AS2 is overexpressed in the bone marrow of patients who acquired adriamycin resistance. There are two downstream targets, miR-520c-3p and S100A4, that may explain the involvement of this lncRNA in chemoresistance. Still, more experimental data is needed (127). Correlations between lncRNAs and the clinical outcome of AML treatment are still not validated in an experimental setting. This is the case of IRAIN, a lncRNA whose low expression is correlated with a worse prognostic after chemotherapy by a higher relapse rate (109). The increased mannosylation (addition of a mannose glycoside) on the membrane proteins is a process through which the AML cells U937 acquire adriamycin resistance. ALG3 alpha-1,3- mannosyltransferase is an enzyme responsible for this process. In ADR/U937 cells, the lncRNA FTX is overexpressed. This lncRNA targets miR-342, thus decreasing its expression and it releases ALG3 mRNA from the RNA interference inhibition (128).

The most commonly course of action in the case of a combinatorial treatment that would involve a lncRNA and a chemotherapeutic would be first sensitizing the cells, with the help of the lncRNA therapeutic inhibition or overexpression, depending on its initial pathological expression level, and then cells are completely eliminated with the help of the chemotherapeutic. In adriamycin-resistant AML cells, if the lncRNA UCA1 is exogenously inhibited, the chemoresistance is impaired, thus the cells are killed by their exposure to adriamycin (112). The knockdown of HOXA-AS2 in adriamycin-resistant AML cells, and further exposure of cells to the same chemotherapeutic resulted in decreased cell viability *in vitro* and tumor growth *in vivo*, thus proving the resensitizing process of AML cells (127).

In order to get a better view of the malignant function of the lncRNAs found to have different expression values between the included cytogenetic risk categories, we researched the literature to find the role of these lncRNAs in other malignancies, as well as their previously reported functions in AML (Table 4).

**Table 4:** General and specific role of lncRNAs with differential expression between different cytogenetic risk categories in AML.

**lncRNAs**	**Function in other malignancies**	**Function in AML**	**References**
DANCR	Oncogenic role, promotes migration, invasion, proliferation	No record found	(129, 130)
PRDM16-DT	Tumor suppressor role	Reported to be overexpressed in Flt3-itd mutation for AML	(102, 131, 132)
SNHG6	Oncogenic role, stimulates cancer cell growth, migration, invasion, cell autophagy	No record found	(133–135)
OIP5-AS1	Oncogenic role, promotes proliferation, maintenance of cell stemness	No record found	(136–138)
SNHG16	Oncogenic role, stimulates proliferation, migration, and invasion	No record found	(139–141)
JPX	Tumor suppressor role	No record found	(142)
FTX	Oncogenic role, stimulates glycolisis, malignant cell proliferation, invasion and migration, poor prognostic	Contributes to MDR in AML-M5	(143–145)
KCNQ1OT1	Oncogenic role, promotes malignant cell proliferation, chemoresistance	No record found	(146, 147)
	Tumor suppressor role, inhibits malignant cell proliferation		(148)
TP73-AS1	Oncogenic role, promotes malignant cell proliferation, invasion and migration, poor prognostic	No record found	(149, 150)
GAS5	Tumor suppressor role, inhibits cell proliferation, invasion, and promotes apoptosis	Mutations in this gene leads to worse prognostic	(151, 152)

*Results from literature review*.

## Long Non-Coding RNAs in Myeloid Malignancies—Analysis of Preclinical *in vivo* Data

The use of mouse models has the advantage of testing *in vivo* the findings initially reported *in vitro*, and potentially using these findings for therapeutic purposes, specifically new therapies for hematological malignancies with no cure other than allogeneic transplantation, as is the case with MDS currently.

ANRIL is a lncRNA which has been reported to reprogram glucose metabolism in AML cells *in vitro* through the induced expression of the Adiponectin receptor (AdipoR1). The *in vivo* experimental model, in which NOD-SCID mice were injected with either shRNA against ANRIL or scrambled shRNA, followed by flow cytometry analysis of their BM sample, validated the *in vitro* data and offered more reliability to the study (153). The same experimental design was used in order to prove the role of HOXA-AS2 overexpression and inhibition of the miR-520c-3p/S100A4 axis in the case of chemoresistance development in AML (127). HOTAIR is a lncRNA which was shown to decrease the *in vivo* tumor formation capacity through the demethylation of the promoter region in the case of the tumor suppressor gene HOXA5 (154). The *in vitro* silencing of the zinc finger antisense 1 (ZFAS1) lncRNA led to a higher percentage of AML apoptotic cells and a slower rate of proliferation of AML cells, while *in vivo* it resulted in the formation of smaller tumors, as compared to untreated mice (94).

We have previously reported the potential role of the lncRNA CCAT2 in the highly amplified 8q24.21 region, with implications for both myeloid malignancies and digestive cancers (55, 129, 155). Fosselteder et al. describe CCAT2 as a lncRNA with genomic localization at the chromosomal region 8q24 amplified in many malignancies (130). This was first described in colon cancer by Ling et al. (131) and later in breast adenocarcinoma by Redis et al. (129, 132). The 8q24 region is highly conserved and encompasses the MYC gene. In myeloid malignancies, a joint study between our institutions was initiated from the observation that CCAT2 is overexpressed in CD34^+^ cells in the bone marrow and in mononuclear cells from peripheral blood isolated from MDS patients, as compared with age-matched healthy individuals. We later discovered that the SNP rs6983267 overlapping CCAT2 is found in two allelic forms that contain either T or G and by using allele specific CCAT2 transgenic mice, confirming this data on patient samples from the US and Romania. *In vivo*, using transgenic mice after 7–9 months, both CCAT2-G mice and CCAT2-T mice had clinical symptoms of an underlying malignancy when compared to the wild-type siblings, having severe leukopenia and lymphocytopenia, moderate anemia and thrombocytosis (55). The CCAT2 role in cancer progression is valid for other types of malignancies and the amplified cancer-associated chromosome 8q24 modulates the Wnt target gene Myc (133), thus affecting the proliferative and self-renewal potential of cancer stem cells (134, 135). Even if this lncRNA was validated in several solid malignancies, including hepatocellular carcinoma, renal cancer and pancreatic cancer (136–138), it has yet to be properly validated in myeloid malignancies. Should it be, it holds great promise to explain the progression of a myelodysplastic syndrome into an acute leukemia.

One of the limitations of the manuscript is the insufficient information presented regarding the potential use of lncRNAs as therapeutics. Very little information has been published thus far regarding the topic, as the mechanisms that underlie their function have yet to be fully elucidated. One direction would be to use these molecules as potential targets. If a long non-coding RNA functions via *cis* regulatory mechanisms, it is extremely challenging to restore its function without expressing at the specific genomic locus (139). However, blocking of the function of long non-coding RNAs could be achieved by several strategies, with the most straightforward approach of downregulation with RNA interference (140). To regain the function of a lncRNA that is lost or downregulated in a myeloid malignancy, the simplest method is to supply with synthetic lncRNA molecules with same function (141). This could be achieved with a non-coding RNA mimic or with its expression vectors (142, 143). When transfected into cells, lncRNA mimics could be processed into a single-strand RNA molecule to target coding genes similar to the endogenous lncRNA, but viable experimental data has yet to be published.

Still, lncRNAs may represent interesting potential targets in AML therapy and monitoring, as they constitute most cellular transcripts and play pivotal roles in hematopoiesis (144, 145). For example, the lncRNA linc00239 (NR_026774.1), 662 nucleotides in length, is upregulated in AML patients, on malignant behaviors and chemosensitivity in AML cells. The presence of linc00239 increases chemoresistance to doxorubicin in AML cells partially by preventing doxorubicin-induced apoptotic cell death and is linked to the activation of the phosphatidylinositol 3-kinase (PI3K)/Akt/mammalian target of rapamycin (mTOR) pathway. Thus, the inhibition of PI3K/Akt/mTOR using 1 μM NVP-BEZ235 (BEZ) abolished the inhibitory effect of linc00239 on chemosensitivity and the preventative effect on doxorubicin-induced cell death (90). Their use could be implemented in risk stratification and prognosis in the clinic. Thus, in a clinical trial setting that enrolled 275 non-M3 AML patients in Taipei, Tsai et al. have shown that higher lncRNA scores were significantly associated with older age and adverse gene mutations. Further, the higher-score patients had shorter overall and disease-free survival than lower-score patients, which were also confirmed in both internal and external validation cohorts (TCGA database) (104). Multivariate analyses linked a lncRNA score to an independent prognosticator in AML, irrespective of the risk based on the ELN classification. In the ELN intermediate-risk subgroup, lncRNA scoring system could well dichotomize the patients into two groups with distinct prognosis. Within the ELN intermediate-risk subgroup, they showed that allogeneic hematopoietic stem cell transplantation could provide better outcomes on patients with higher lncRNA scores. Through a bioinformatics approach, they identified high lncRNA scores correlated with leukemia/hematopoietic stem cell signatures. Thus, incorporation of lncRNA scoring system in the 2017 ELN classification can improve risk-stratification of AML patients and help in clinical decision-making.

## Conclusion

There are several lncRNAs that have been implicated in several roles in AML. ANRIL, RP11-732M18.3, LNC_104449, CASC15, CCDC26, DANCR, LINC00467, MIR9-3HG, AL035071.1, AP001042.1, RP11-395P13.6-001, FAM30A, H22954, LEF1-AS1, LINC00152, LINC00239, LINC00265, LINC00319, LINC00504, LINC00899, LINC00926, LNC177417, LRRC75A-AS1, MALAT1, NEAT1, PANDAR, PILNA, RP11-222k16.2, RP11-305O.6, SBF2-AS1, TUG1, UCA1, ZFAS1 have potential oncogenic roles in AML. RP11-704M14.1, SFMBT2-4:1, IRAIN, TET2 function as tumor suppressors in AML.

There are two lncRNAs with both oncogenic and tumor suppressor roles in AML. MEG3 is upregulated in AML-M3, but in AML cell lines is downregulated. CRINDE is downregulated in AML-M3 and upregulated in AML bone marrow samples and cell lines.

There are lncRNAs with specific expressions for each FAB AML subtype. H19 is upregulated in M1-2 AML. LINC00877, RP11-84C10.2, RP11-848P1.3, ZNF667-AS1 are downregulated in M3 AML, and LINC02082-201, AC009495.2 are upregulated in M3 AML. HOTTIP, SNHG5, CCAT1 are upregulated specifically in M4-5 AML. CCAT1, PVT1, and XLOC_109948 are overexpressed in AML patients bearing increased risk mutations. HOTAIR is upregulated only in stem cells from bone marrow samples of AML patients.

Our original analysis of TCGA data on AML revealed that PRDM16-DT is downregulated, and DANCR is upregulated in poor cytogenetic prognostic AML samples compared with normal cytogenetic risk. OIP5-AS1 is downregulated in both poor and favorable prognostic compared with normal cytogenetic risk. SNHG6 is underexpressed in poor prognostic compared with intermediate cytogenetic risk and overexpressed in poor prognostic compared with favorable prognostic. SNHG16, JPX, FTX, KCNQ1OT1 are upregulated in AML samples with favorable risk as compared with normal risk. GAS5 is overexpressed in poor prognostic compared with favorable prognostic, whereas TP73-AS1 is upregulated specifically in poor prognostic patients, being overexpressed in comparison with favorable and normal cytogenetic risk. These lncRNAs have the potential of becoming future biomarkers of cytogenetic risk, however they still need further validation on bone marrow samples, peripheral blood from another cohort, along with additional gene expression analysis methods.

The potential of lncRNAs in their use as biomarkers or therapeutic targets is still in its infancy. More knowledge has to be gained on their established molecular interactions, biological roles, standardization of isolation and gene expression analysis. As follows, more data is needed to fully introduce the idea of clinical uses of lncRNAs in the clinical evaluation or treatment of AML, especially in a large cohort non-interventional clinical trial that would be able to compare the sensitivity and specificity of currently applied diagnostic/prognostic methods.

## Author Contributions

A-AZ did the bioinformatics analysis. CT and A-AZ wrote the manuscript. IS critically revised the manuscript. GC and IB-N contributed to the design of the manuscript and supervised the project.

### Conflict of Interest

The authors declare that the research was conducted in the absence of any commercial or financial relationships that could be construed as a potential conflict of interest.

## Abbreviations

GAS5: Growth Arrest Specific 5
AML: acute myeloid leukemia
ANRIL: antisense RNA in the INK4 locus
BM: bone marrow
CASC15: cancer susceptibility 15
CCAT1: colon cancer associated transcript 1
CRNDE: colorectal neoplasia differentially expressed
DANCR: differentiation antagonizing non-protein coding RNA
FTX: FTX transcript, XIST regulator
H19-H19: imprinted maternally expressed transcript
HOTAIR: HOX transcript antisense RNA
HOTTIP: HOXA distal transcript antisense RNA
IRAIN: IGF1R antisense imprinted non-protein coding RNA
JPX-PX transcript: XIST activator
KCNQ1OT1: KCNQ1 opposite strand/antisense transcript 1
LEF1-AS1: lymphoid enhancer binding factor 1 antisense RNA 1
MALAT1: metastasis associated lung adenocarcinoma transcript 1
MAPRE1: microtubule associated protein RP/EB family member 1
MEG3: maternally expressed 3
NEAT1: nuclear paraspeckle assembly transcript 1
NK cells: natural killer cells
NTRK3: neurotrophic receptor tyrosine kinase 3
OIP5-AS1: Opa interacting protein 5 antisense RNA 1
PANDAR: promoter of CDKN1A antisense DNA damage activated RNA
PRDM16-DT: PR/SET domain 16 divergent transcript
SBF2-AS1: SET binding factor 2-antisense RNA 1
SNHG: small nucleolar RNA host gene
SOX4: SRY-Box 4
TET2: tet methylcytosine dioxygenase 2
TP73-AS1: tumor protein P73 antisense RNA 1
TUG1: Taurine up-regulated 1
UCA1: urothelial cancer associated 1
ZFAS1: ZNFX1 antisense RNA 1.
